# Joint fluid interleukin-6 combined with the neutral polymorphonuclear leukocyte ratio (PMN%) as a diagnostic index for chronic periprosthesis infection after arthroplasty

**DOI:** 10.1186/s10195-023-00712-8

**Published:** 2023-07-04

**Authors:** Xudong Su, Bo Zhu, Leilei Qin, Jianye Yang, Li Wei, Zhenghao Xu, Keyu Wei, Jiayi Wang, Li Chen, Chen Zhao, Cheng Chen, Wei Huang, Yan Xiong, Ning Hu

**Affiliations:** 1 Department of Orthopedics, The First Affiliated Hospital of Chongqing Medical University, Chongqing, 400016 China; 2 Laboratory of Orthopedics, Chongqing Medical University, Chongqing, 400016 China; 3 Department of Orthopedics, Daping Hospital, Army Medical University, Chongqing, China

**Keywords:** Joint fluid, Interleukin 6, Neutral polymorphonuclear leukocytes (PMN%), Periprosthetic infection, Diagnosis

## Abstract

**Background:**

The diagnosis of periprosthetic joint infection (PJI) remains a challenge in clinical practice. Many novel serum and joint fluid biomarkers have important implications for the diagnosis of PJI. The presented study evaluated the value of joint fluid interleukin-6 (IL-6) combined with the neutral polymorphonuclear leukocyte (PMN%) ratio for chronic PJI diagnosis after arthroplasty.

**Materials and methods:**

Sixty patients with chronic PJI or aseptic failure who underwent hip or knee revision from January 2018 to January 2020 in our department were included in this retrospective study. According to the 2013 MSIS diagnostic criteria, the 60 patients were divided into a PJI group and a non-PJI group (30 patients per group). We collected the joint fluid before surgery and determined the level of IL-6 and the PMN% by ELISA, and the differences between the two groups were compared. The diagnostic efficacy of joint fluid IL-6 combined with PMN% in chronic PJI was analyzed using a receiver operating characteristic curve (ROC curve).

**Results:**

The diagnosis of PJI using joint fluid IL-6 combined with PMN% presented an area under the curve of 0.983, which was more accurate than the areas under the curve for diagnosis using IL-6 and PMN% individually (0.901 and 0.914, respectively). The optimal threshold values for IL-6 and PMN% were 662.50 pg/ml and 51.09%, respectively. Their sensitivity and specificity were 96.67% and 93.33%, respectively. The accuracy of the diagnosis of PJI was 95.00%.

**Conclusions:**

Joint fluid IL-6 combined with PMN% can be used as an auxiliary method to detect chronic infection around the prosthesis after hip/knee arthroplasty.

**Level of evidence:**

Patients who underwent hip/knee revision at the First Hospital of Chongqing Medical University for periprosthetic infection or aseptic failure of the prosthesis after hip/knee arthroplasty from January 2018 to January 2020 were included.

*Trial registration* This study was approved by the ethics committee of the First Hospital of Chongqing Medical University on September 26, 2018 (local ethics committee number: 20187101) and registered with the China Clinical Trials Registry (registration number: ChiCTR1800020440) with an approval date of December 29, 2018.

## Introduction

Total joint arthroplasty (TJA) is an effective treatment modality for end-stage bone and joint disease [1, 2]. Although there have been significant improvements in preventing periprosthetic joint infection (PJI), infection remains a significant challenge and a common cause of failure after TJA [3, 4]. The reported incidence of PJI is still between 0.5 and 2.0%, and it has become the leading cause of total knee arthroplasty failure and the third leading cause of total hip arthroplasty revision [5, 6]. In recent years, although there have been many advances in the diagnosis of PJI, distinguishing between post-arthroplasty infection and aseptic failure remains difficult in some cases. Therefore, establishing an accurate and timely diagnostic strategy is crucial for the management of PJI [7].

Many novel serum and joint fluid biomarkers, such as CD14, TREM-1, and TLR2, have shown promising potential in the diagnosis of PJI [8–12]. Qin et al. identified D-dimer as a valuable biomarker for the detection of chronic PJI. The combination of serum D-dimer and C-reactive protein (CRP) resulted in increased sensitivity compared to diagnosis based on serum D-dimer and CRP separately [1]. However, the detection techniques used for many biomarkers are not readily available in many hospitals, and there are varying degrees of controversy among different studies, making it difficult for the detection results to be widely used in clinical work. IL-6 is a cytokine produced by a variety of cells, including monocytes and macrophages, in response to immune responses, and can be significantly upregulated in pathogenic infection [13]. Neutrophils are the most abundant innate immune cells in the circulating blood, and they are one of the first lines of defense of the immune system against infections [8]. The diagnostic value of serum IL-6 in PJI was confirmed by previous studies, but its diagnostic sensitivity and specificity are not ideal [14].

Considering that the level of inflammatory markers in the joint fluid better reflects local inflammation [15], in this study, we tried to use IL-6 combined with PMN% in joint fluid to effectively identify the exact condition (chronic PJI or aseptic failure) in patients undergoing revision surgery after total joint arthroplasty and to obtain the threshold values for determining chronic PJI. This study also aimed to provide a reference for the diagnosis of chronic PJI.

## Materials and methods

### Study design and inclusion and exclusion criteria

This study was approved by the ethics committee of the First Hospital of Chongqing Medical University on September 26, 2018 (local ethics committee number: 20187101) and registered with the China Clinical Trials Registry (registration number: ChiCTR1800020440) with an approval date of December 29, 2018. Patients who underwent hip/knee revision at the First Hospital of Chongqing Medical University for periprosthetic infection or aseptic failure of the prosthesis after hip/knee arthroplasty from January 2018 to January 2020 were included. Inclusion criteria: age > 18 years; periprosthetic infection or aseptic failure of the prosthesis after initial replacement, with a recommendation for second-stage revision surgery; no other infection during the 2-year follow-up after hip/knee replacement. Exclusion criteria: (1) inflammatory arthritis, such as rheumatoid arthritis; (2) patients who had used antibiotics within the previous 2 weeks; (3) a history of petechiae, prosthetic heart valves, or hypercoagulation; (4) heavy smokers or patients with malignancy; (5) acute infections [16, 17].

The diagnostic criteria for chronic PJI were the isolation of pathogens from two or more separate tissue or fluid samples from around the involved prosthetic joint and the presence of at least four of the following six criteria: (1) elevated erythrocyte sedimentation rate (ESR) and CRP; (2) elevated joint fluid white blood cell count; (3) elevated joint fluid percent neutrophils (PMN%); (4) pus from the involved joint; (5) the isolation of microorganisms from periprosthetic joint tissue or fluid cultures; (6) periprosthetic histopathological specimens with a mean neutrophil count of 5 in five high-powered fields under high-powered microscopy. Patients were divided into a periprosthetic infection group (in which the patients had symptoms of infection, such as pain, that were present for more than 6 weeks after the implantation of the prosthesis) and a non-infected (non-PJI) control group [10].

### Sample collection and determination

Clinical data relevant to the study included medical histories and laboratory examinations from patients undergoing revision surgery. The medical history included the patient’s age, gender, BMI, previous surgery, functional status of the affected limb at the current admission, presence of nocturnal or resting pain, presence of fever or a definite history of infection and comorbidities, and physical examination (whether the joint skin temperature was elevated, presence of redness, swelling, heat and pain, presence of sinus tract or pus). Laboratory examinations included serum CRP, ESR, PMN%, and joint fluid IL-6 testing on admission. In addition, all patients discontinued antibiotics for 2 weeks before arthrocentesis, and joint fluid was obtained by arthrocentesis at admission or preoperatively, with cell sorting and pathogenic microorganism culture. Histopathology and pathogenic microorganism culture of at least three periprosthetic tissues were performed intraoperatively. Microbial cultures of all specimens included aerobic, anaerobic, fungal, and prolonged cultures.

Immediately after admission, 1–2 ml of joint fluid were collected. 2 h after collection, all cellular and particulate components were removed by centrifugation at 1000 rpm for 10 min. The levels of IL-6 and PMN% in the joint fluid were measured using an IMMULITE 1000 immunoassay system (Siemens Healthcare, Erlangen, Germany). Serum ESR and CRP were measured by blood collection at admission and 1 day before surgery, respectively.

### Statistical analysis

Categorical variables were analyzed using SPSS version 25 software (IBM Corp., Armonk, NY). Continuous variables were expressed as mean ± standard deviation, and categorical variables were expressed as counts and percentages. The independent samples *t*-test was used for measures that conformed to a normal distribution, and the Mann–Whitney *U* test was used to compare the differences between the two groups for measures that did not conform to a normal distribution. Correlations between variables were analyzed using Pearson’s correlation coefficient. MedCalc 15.2.2 software (MedCalc Software, Ostend, Belgium) was used to analyze the receiver operating characteristic (ROC) curves and the area under the curve (AUC) values. Youden’s* j* statistic was used to determine the optimal cut-off value for the diagnosis of chronic PJI. The sensitivity, specificity, accuracy, positive predictive value (PPV), negative predictive value (NPV), positive likelihood ratio, negative likelihood ratio, and diagnostic odds ratio (DOR) were calculated for two serum markers (CRP, ESR) and joint fluid IL-6. *p* < 0.05 was considered statistically significant.

## Results

Table 1 shows the demographics of the two groups. Thirty patients who were judged as having chronic PJI based on the MSIS 2013 diagnostic criteria were matched 1:1 with non-PJI patients according to age, sex, and body mass index (BMI). Thirty non-PJI patients were included in the control group (Table 1). The differences in the general data between the two groups, including age, weight, height, BMI, gender, and joint type, were not statistically significant (*P* > 0.05).

**Table 1:** Demographic data for the study population

Index	Non-PJI (*n* = 30)	PJI (*n* = 30)	*P* value
Age	70.040 ± 6.554	64.7200 ± 13.752	0.09
Weight (cm)	60.400 ± 9.917	60.160 ± 12.821	0.94
Height (kg)	157.920 ± 5.943	161.160 ± 8.163	0.12
BMI	24.230 ± 3.854	23.170 ± 4.640	0.38
Gender			0.77
Female	17 (28.3%)	14 (23.3%)	
Male	13 (21.7%)	16 (26.7%)	
Joint type			0.78
Knee	16 (26.7%)	12 (20.0%)	
Hip	14 (23.3%)	18 (30.0%)	

The levels of IL-6 and PMN% in joint fluid and of ESR and CRP in serum in both groups are shown in Table 2. The IL-6 concentration in joint fluid in the PJI group was 1154.50 (893.00) pg/ml, which was significantly higher than that in the non-PJI group, 390.50 (394.50) pg/ml (*P* < 0.001). The PMN% of joint fluid in the PJI group, 69.71% (25.85%), was significantly higher than that in the non-PJI group: 40.06% (22.49%) (*P* < 0.001). Serum ESR and CRP concentrations were not statistically different in the PJI (ESR: 35.00 (33.50) mm/h; CRP: 19.10 (18.40) mg/L) and non-PJI (ESR: 30.00 (16.00) mm/h; CRP: 16.40 (19.79) mg/L) groups (*P* > 0.05).

**Table 2:** Analysis of inflammatory markers in patients with periprosthetic infection and aseptic revision arthroplasty

Index	Non-PJI (*n* = 30)	PJI (*n* = 30)	*P* value
ESR (mm/h)	30.00 (16.00)	35.00 (33.50)	0.403
CRP (mg/L)	16.40 (19.79)	19.10 (18.40)	0.162
IL-6 (pg/ml)	390.50 (394.50)	1154.50 (893.00)	< 0.001
PMN%	40.06 (22.49)	69.71 (25.85)	< 0.001

*CRP* C-reactive protein, *ESR* erythrocyte sedimentation rate

**Fig. 1:**
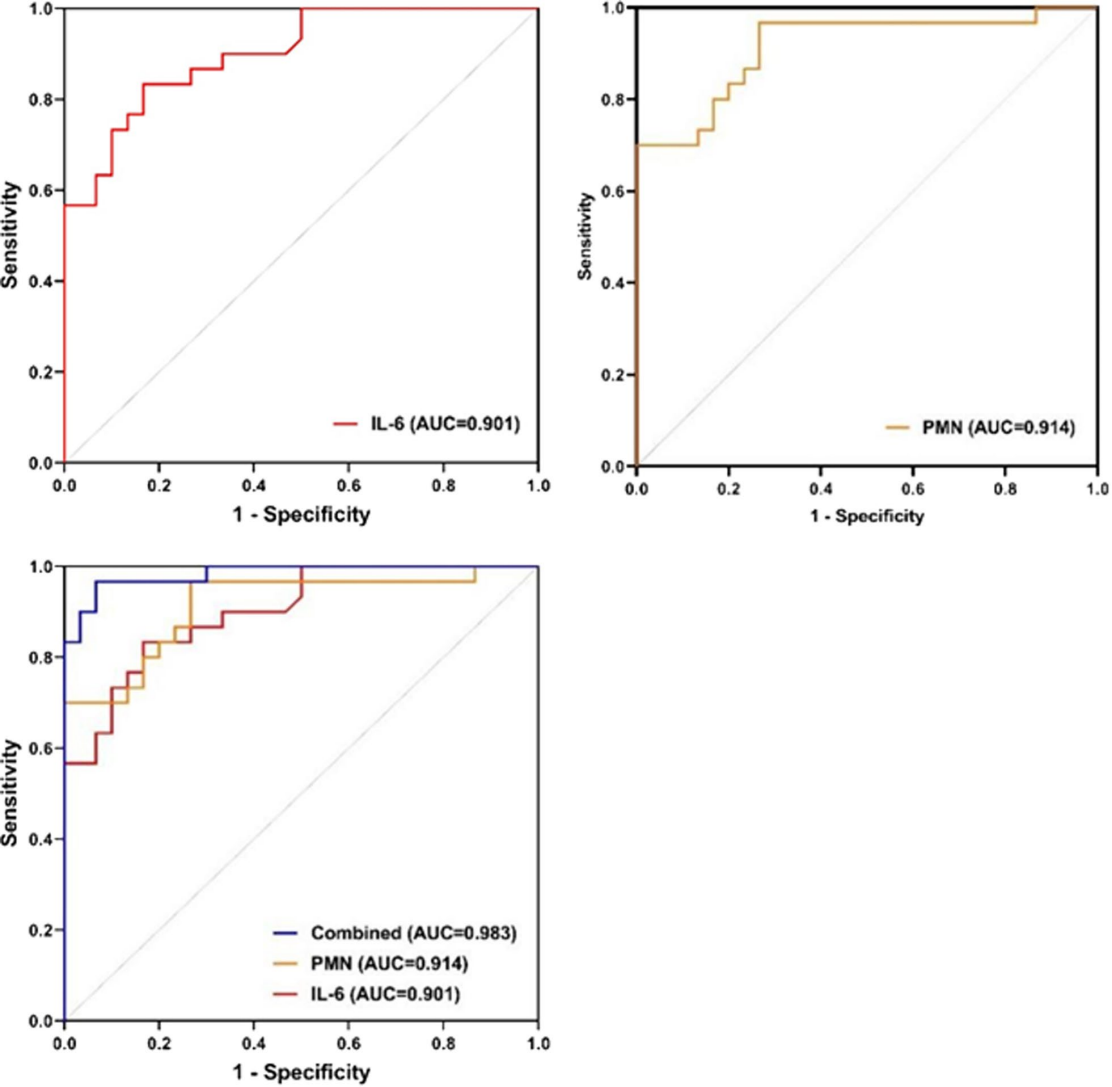
ROC curves showing the PJI predictive value of IL-6 and PMN%. *ROC* receiver operating characteristic, *PJI* periprosthetic joint infection

To assess the predictive value of joint fluid IL-6 and PMN% for chronic PJI, we developed a ROC diagnostic analysis model (Fig. 1), determined the optimal cut-off value from the ROC curve, and calculated the AUC to calculate the specificity, sensitivity, and accuracy of these markers for the diagnosis of chronic PJI. The AUC for joint fluid IL-6 was 0.901 (0.827 to 0.975) and the AUC for PMN% was 0.914 (0.841 to 0.988). The AUC for IL-6 combined with PMN% for the diagnosis of chronic PJI was 0.983 (0.959 to 1.000). The sensitivity for diagnosing chronic PJI was 83.33% (64.55 to 93.69%), the specificity was 83.33% (64.55 to 93.69%), and the accuracy was 83.33% when the critical value of joint fluid IL-6 was 662.50 pg/ml. When the critical value was 51.09%, the sensitivity of PMN% for detecting PJI was 96.67% (80.95 to 99.83%), the specificity was 73.33% (53.83 to 87.02%) and the accuracy was 85.00%. Further evaluation of the combined diagnostic value of joint fluid IL-6 combined with PMN% for chronic PJI showed a sensitivity of 96.67% (80.95 to 99.83%), specificity of 93.33% (76.49 to 98.84%), and accuracy of 95.00% for the diagnosis of chronic PJI (Table 3).

**Table 3:** Sensitivity, specificity, PPV, NPV, and accuracy of inflammatory markers

Index	AUC (95% CI)	Cutoff value (pg/ml or %)	Sensitivity (95% CI) (%)	Specificity (95% CI) (%)	Accuracy (%)
IL-6	0.901 (0.827 to 0.975)	662.50	83.33 (64.55 to 93.69)	83.33 (64.55 to 93.69)	83.33
PMN%	0.914 (0.841 to 0.988)	51.09	96.67 (80.95 to 99.83)	73.33 (53.83 to 87.02)	85.00
PMN% + IL-6	0.983 (0.959 to 1.000)	/	96.67 (80.95 to 99.83)	93.33 (76.49 to 98.84)	95.00

*PPV* positive predictive value, *NPV* negative predictive value, *AUC* area under the curve, *CI* confidence interval

Table 4 shows the relevant diagnostic features of the inflammatory indicators IL-6 and PMN% in the synovial fluid. The PPV and NPV of IL-6 in synovial fluid for the diagnosis of chronic PJI were 83.33 and 83.33%, respectively (for a cutoff value of 662.50 pg/ml). The PPV and NPV of PMN% for the diagnosis of PJI were 78.38 and 95.65%, respectively (for a cutoff value of 51.09%), and the PPV and NPV of IL-6 combined with PMN% were 93.55 and 96.55%, respectively. The positive likelihood ratio, negative likelihood ratio, and diagnostic odds ratio (DOR) for IL-6 combined with PMN% in the patients’ synovial fluid were 14.50, 0.04, and 406, respectively. The positive likelihood ratio, negative likelihood ratio, and DOR for IL-6 were 5, 0.2, and 25, respectively, and the positive likelihood ratio, negative likelihood ratio, and DOR for PMN% were 3.625, 0.045, and 80.56, respectively.

**Table 4:** Performance of IL-6 and PMN% in the diagnosis of PJI

Test	IL-6	PMN%	PMN% + IL-6
Sensitivity (%)	83.33%	96.67%	96.70%
Specificity (%)	83.33%	73.33%	93.33%
PPV (%)	83.33%	78.38%	93.55%
NPV (%)	83.33%	95.65%	96.55%
LR +	5	3.625	14.50
LR −	0.2	0.045	0.04
DOR	25	80.56	406

*CRP* C-reactive protein, *ESR* erythrocyte sedimentation rate, *PJI* periprosthetic joint infection, *PPV* positive predictive value, *NPV* negative predictive value, *LRs* likelihood ratio, *DOR* diagnostic odds ratio

## Discussion

The clinical symptoms and laboratory markers that patients with chronic PJI may show are not obvious, which greatly increases the difficulty of diagnosing chronic PJI [3, 18–20]. Therefore, it is crucial to seek means that can accurately diagnose infection. In recent years, with the exploration of novel inflammatory markers and the study of prosthetic ultrasonic lysis culture and molecular diagnostic techniques, the diagnostic accuracy of PJI has been greatly improved [21, 22]. Considering that serum inflammatory markers may be affected by concomitant inflammation in other organs and systems, the assessment of local markers of the affected joint is more valuable for the diagnosis of PJI [7, 23, 24]. High levels of IL-6 in body fluids are associated with acute localized bacterial infections and are an anti-inflammatory factor and mediator of the infection response [25, 26]. Related studies have shown that serum CRP levels are elevated 4–6 h after IL-6 stimulation, suggesting that IL-6 is a relatively sensitive biomarker of early immune responses [27]. PMN is an important part of the human body and plays an important role in resisting microbial invasion and promoting the occurrence, development, and resolution of inflammation. Elevated synovial fluid PMN% has been identified as a useful marker for the diagnosis of PJI in previous studies [28]. In this study, we measured joint fluid IL-6 and PMN% levels in 60 patients to explore the value of joint fluid IL-6 combined with PMN% in distinguishing chronic PJI from aseptic failure, and we found that joint fluid IL-6 combined with PMN% has a high recognition accuracy (95.00%) for PJI.

Previous studies have demonstrated that IL-6 in joint fluid and blood is useful in the diagnosis of chronic PJI but cannot be used as the sole diagnostic criterion [29]. A prospective case–control study showed that serum IL-6 is a valuable and more accurate index for the diagnosis of PJI than ESR and CRP, with a sensitivity and specificity of 0.97 and 0.91, respectively, for the diagnosis of PJI [30]. A systematic evaluation conducted in 2018 explored the diagnostic accuracy of serum, synovial, and tissue tests for PJI and showed that joint fluid IL-6 had a good specificity (0.971) but varying degrees of decreased sensitivity (0.875) [31]. This result is similar to the present study, which found that the sensitivity of joint fluid IL-6 for chronic PJI was 83.33% and its diagnostic specificity was 83.33%, but the diagnostic accuracy of joint fluid IL-6 combined with PMN% for chronic periprosthetic infection after joint replacement was 95.00%, indicating that joint fluid IL-6 combined with PMN% has a higher diagnostic value for chronic PJI. Kai Xie et al. [25] also found that the AUC of joint fluid IL-6 levels for the diagnosis of PJI was higher than that of serum IL-6 levels, with an AUC of 0.96, a sensitivity of 91%, a specificity of 90%, and a diagnostic threshold of 2300 pg/ml. The detection of IL-6 with a threshold of 662.50 pg/ml in combination with the detection of PMN% with a threshold of 51.09% was found to provide the best discrimination between chronic PJI and aseptic failure of the prosthesis, yielding an accuracy of 95%. Its ability to significantly improve the accuracy of PJI diagnosis gives it superior clinical diagnostic significance.

This study has some limitations. First, there is currently a lack of a real gold standard for diagnosing PJI. We used the 2013 MSIS diagnostic criteria as the basis for grouping in this study, and some patients in the non-PJI group may have been false-negative patients, but this is a situation faced by all studies evaluating the diagnosis of PJI infection. Second, the sample size of our study was small. However, as an initial test, the current study has shown promising results, so a larger multicenter study could be conducted to further validate the findings. Finally, patients on antibiotics for two weeks before surgery were excluded from this study to eliminate confounding factors. However, this may have created testing prerequisites that differ from those used in clinical practice, limiting the generalizability of the results of this study.

Our study found significantly elevated IL-6 and PMN% values in the synovial fluid of PJI patients. Based on the MSIS criteria, IL-6 combined with PMN% had higher sensitivity and specificity for the diagnosis of chronic PJI. From the above analysis, we believe that IL-6 combined with PMN% in synovial fluid is a promising diagnostic index for PJI and may be included in the diagnostic criteria for PJI in the future. This combination of joint synovial fluid biomarkers can improve the accuracy of PJI diagnosis, providing clinicians with a more timely and accurate diagnosis of PJI, and bringing benefits to patients. Therefore, more research that focuses on finding biomarkers with high accuracy, cost-effectiveness, and feasibility is needed.

## Conclusion

In this study, it was shown that the combination of joint fluid IL-6 and PMN% can be used as a specific molecular marker for the diagnosis of chronic PJI, and the most appropriate thresholds for IL-6 and PMN% were found to be > 662.50 pg/ml) and > 51.09%, respectively. This combination is more sensitive and specific in distinguishing aseptic failure after arthroplasty from chronic PJI than IL-6 or PMN% in joint fluid alone. However, there are few clinical examples of the combined use of joint fluid IL-6 and PMN% for the diagnosis of PJI, so more evidence is needed to support the use of joint fluid IL-6 combined with PMN% as a valid reference indicator for diagnosing PJI and determining the timing of prosthetic reimplantation.

## Data Availability

The data that support the fundings of this study are available from the corresponding author, Ning Hu, upon reasonable request.

## Abbreviations

PJI: Prosthetic joint infection
IL-6: Interleukin-6
ROC: Receiver operating characteristic
CRP: C-reactive protein
ESR: Erythrocyte Sedimentation Rate
SF: Synovial fluid
CI: Confidence interval
PPV: Positive predictive value
NPV: Negative predictive value
AUC: Area under the curve
MSIS: Musculoskeletal Infection Society
MR: Mannose-receptor
G-CSF: Granulocyte colony-stimulating
TNF-α: Tumor necrosis factor α
IFN-γ: Interferon γ
